# Language or motor: reviewing categorical etiologies of speech sound disorders

**DOI:** 10.3389/fpsyg.2015.01708

**Published:** 2015-11-04

**Authors:** Kelly Farquharson

**Affiliations:** Department of Communication Sciences and Disorders, Emerson CollegeBoston, MA, USA

**Keywords:** speech sound disorders, speech development, motor ability, language development, speech language therapy

Children with speech sound disorders (SSDs) exhibit marked weakness with accurate production of age-appropriate speech sounds (Lewis et al., 2006). For some of these children, the etiology of the SSD is clear (e.g., cleft palate, a genetic syndrome, or hearing loss). For others, the cause of their aberrant speech development is unknown; this type of SSD is “functional.” Functional SSDs may eventually remediate after a course of treatment, but may also persist into adolescence or event adulthood (Felsenfeld et al., 1994). Regardless of the outcome, the underlying construct that contributes to this disorder remains elusive. The extant literature is comprised of two primary categorical constructs used to explain functional speech sound disorders: language-based deficits and motor-based deficits. Undeniably, all speech productions are both linguistic (speech sounds, meanings of words, syntax of context, etc.) and motoric (the muscle movement of the speech articulators—lips, tongue, jaw, soft palate, etc.) in nature. However, there are certainly competing theories that suggest language (Raitano et al., 2004; Sutherland and Gillon, 2005; Lewis et al., 2006; Preston and Edwards, 2007; Anthony et al., 2011) or motor (Webster et al., 2005; Newmeyer et al., 2007; Peter and Stoel-Gammon, 2008; Visscher et al., 2010; Redle et al., 2015) to be the predominant causal mechanism for persistent deficits in speech production abilities. I argue that the relation between motoric and linguistic ability is likely complimentary, rather than starkly categorical.

Although on some levels, the distinction between these two constructs may seem trivial, it is clinically prudent to consider. Presently, many school districts across the country deny services to children who have “just” articulation (i.e., motor-based) impairments. Interestingly, even children with “just” an articulation impairment have been reported to experience academic difficulties, even once the child has remediated the speech production error (Raitano et al., 2004; Farquharson, 2012, 2015). Specifically, difficulties with reading, spelling, and phonological awareness persist often throughout schooling. Studies have supported that adults with a history of speech sound disorders have more often repeated a grade in school than adults with no history. Interestingly, some children with SSD who have spelling difficulties exhibit similar error patterns within their spelling as they do in their speech (e.g., substitution of a particular phoneme, such that a word like “rain” may be spelled “wain”). Such reports would suggest that speech sound disorders are not strictly motoric in nature. However, as a field, we remain unclear on the extent to which motoric deficits contribute to SSDs and the relationship between language ability and motoric ability. That is, although the speech articulators are not independently achieving correct placement for age-appropriate speech sounds, it is often the case that the child is *able* to correctly move the articulators, but *does not* do so in connected speech. Some research indicates that this discrepancy is related to phonological representations, or the process by which linguistic/phonological information is stored within memory.

Phonological representations may be difficult to access for children with speech sound disorders due to underlying linguistic or cognitive deficits (Larrivee and Catts, 1999; Sutherland and Gillon, 2005; Farquharson, 2012, 2015). The development of phonological representations requires specification of phonological details as well as organization of the segments of a word (Swan and Goswami, 1997). For children who have phonological weaknesses, such as those with speech sound disorders and/or dyslexia these representations do not develop properly. As a result, activities that require repeated access to these representations—reading, speaking, spelling—are difficult or impossible (Sutherland and Gillon, 2005; Preston and Edwards, 2007). Phonological forms that are more complex, have more syllables, or are less familiar will be particularly difficult. This is educationally relevant because children encounter substantial amounts of new vocabulary as they progress through school. For children with speech sound disorders, their ability to access, store, and use those words is circumscribed by their phonological deficits. However, there is a separate body of work that has provided substantial evidence that children with speech sound disorders exhibit motoric weaknesses.

Motor ability has been measured in children with speech sound disorders and has examined oral motor, fine motor, and gross motor abilities. For instance, Peter and Stoel-Gammon reported central timing deficits in children with SSD, as evidenced by weaknesses in non-word repetition, clapping imitation, and paced tapping. However, in that study, the researchers examined language skills but did not report them or use them for covariates in analyses. As such, the contribution of language, especially to non-word repetition skills, is not considered. Recently, Redle et al. (2015) reported neuroimaging and behavioral data examining the motoric abilities of children with SSDs. Their results revealed that children with SSDs exhibited weaker oral and fine motor skills compared to typically developing peers. Similar to other investigations of children with SSDs (Farquharson, 2012, 2015), Redle and colleagues found that persistent SSD group performed within the average range for language and cognitive skills, but still significantly differ from their peers. This was strong evidence to support a motoric deficit in children with an otherwise functional SSD. One caveat to this study is that the researchers gathered information regarding the children's classroom performance via parent survey. It would be interesting to gather these data directly from the classroom teacher and examine how the child is truly performing academically. It remains unclear how these “subclinical” linguistic and cognitive deficits interact with the motoric weaknesses.

In my opinion, it is very likely that language and motor have an intricate relationship in terms of speech production. For instance, a young child who exhibits difficulty with speech sound production due to motor-based deficits may eventually persist with the speech sound production errors as a result of eventual language deficits. That is, the motor deficits may have “snow-balled” into language deficits after repeated incorrect production of meaningful linguistic units. Over time, those incorrect productions may result in incorrect phonological representations—this causes difficulties with language and literacy-based skills. Certainly, this particular scenario needs empirical support. However, from my perspective, this seems to be a logical and plausible explanation of the relation between language and motor for children with speech sound disorders.

Collectively, research supports that children with SSDs perform below their typically developing peers on measures requiring linguistic and motoric output. Thus, it is possible that the contributions of motor and language to speech production are not disparate, but are dynamically complimentary (see Nip et al., 2009; Iverson, 2010, for reviews). To date, there is not one comprehensive investigation of both of these constructs within the same population of children with SSDs. Such a study would substantiate the relationship between language and motor and potentially ascertain the direction of said relationship.

In conclusion, it is evident that future work is necessary to better conceptualize the underlying mechanisms related to speech sound disorders—theoretical or otherwise. Such work will help to improve the quality of both assessment and treatment of this population of children. Further, it is hoped that this line of work will provide policy-makers and administrators with the evidence necessary to make appropriate decisions regarding service provision. It is unjust to regard any form of communication impairment as “just” a deficit that a child should deal with for life.

## Conflict of interest statement

The author declares that the research was conducted in the absence of any commercial or financial relationships that could be construed as a potential conflict of interest.
